# Removal of Implant and New Rehabilitation for Better Esthetics

**DOI:** 10.1155/2018/9379608

**Published:** 2018-06-06

**Authors:** Wilson Matsumoto, Victor Garone Morelli, Rossana Pereira de Almeida, Alexandre Elias Trivellato, Cássio Edvard Sverzut, Takami Hirono Hotta

**Affiliations:** Dental School of Ribeirão Preto, Dental Materials and Prosthodontic Department, University of São Paulo, Ribeirão Preto, SP, Brazil

## Abstract

Tooth loss can result in loss of facial esthetics, in addition to its effect on mastication, swallowing, and speech. Adequate planning is required in order for the prosthetic treatment of the teeth and implants to be successful. Here, we present a clinical case demonstrating that improper positioning of an implant can make prosthetic rehabilitation unfeasible, necessitating new surgical and rehabilitation planning to achieve the desired esthetics. The patient had a missing right lateral incisor and cuspid, and a buccally directed implant. The preferred treatment regimen involved the removal of this implant and placement of another one more properly positioned distally and three dimensionally. The repositioning of the implant made rehabilitation treatment with a mesial cantilever possible. Due to esthetic considerations, gingival conditioning in the cantilever region was performed in the temporary prosthesis phase. The changes adopted in planning the surgery and rehabilitation resulted in good esthetics as well as functional outcomes.

## 1. Introduction

It is known that tooth loss negatively affects swallowing, chewing, phonetics, and mandibular posture, as well as the patient's facial esthetics, all of which may lead to social and emotional discomfort. Consequently, when the treatment option involves the placement of implants, appropriate care must be taken to ensure the accurate three-dimensional placement of the implant [1], and that the implant is in harmony with the opposing and adjacent teeth [2], especially when esthetic areas are involved [3] where there is the necessity to precisely evaluate the existence of bone and soft tissue deficiencies.

The papillae adjacent to the single-unit crown should mimic those of a healthy tooth, in both height and embrasure fill, and the midbuccal gingival margins should harmonize with those of the adjacent teeth [3, 4]; however, the progressive involution of the alveolar bone begins following tooth loss, and it can be accompanied by a marked reduction in both the quality and quantity of hard and soft tissues [5], and the final result may not be acceptable from an esthetic point of view.

As a dental procedure, diagnostic waxing-up can be used to visualize the final result of prosthetic treatment [1], thereby allowing an analysis of the emergency profile and the shape and size of the teeth, as well as the surgical guide to check the correct position of the implant during the placement [6, 7]. The use of techniques, such as computerized guided surgery, stereolithographs, and three-dimensionally printed surgical guides, has made the results of implant prosthesis treatment better and more predictable [8].

However, despite all prior care, the implant can be placed in an improper position, making prosthetic restoration impracticable [9]. In this situation, removal of the implant or its repositioning have been proposed [10], and several types of procedures can be used to solve this problem [9, 11–13]. In this context, the professional should select the option that best suits the specific case, seeking the preservation of soft tissues and the patient's comfort and welfare. Here, we present a case in which the removal of a buccally positioned implant was required, and we demonstrate a new surgical procedure and successful prosthetic treatment.

## 2. Case Report

**Figure 1:**
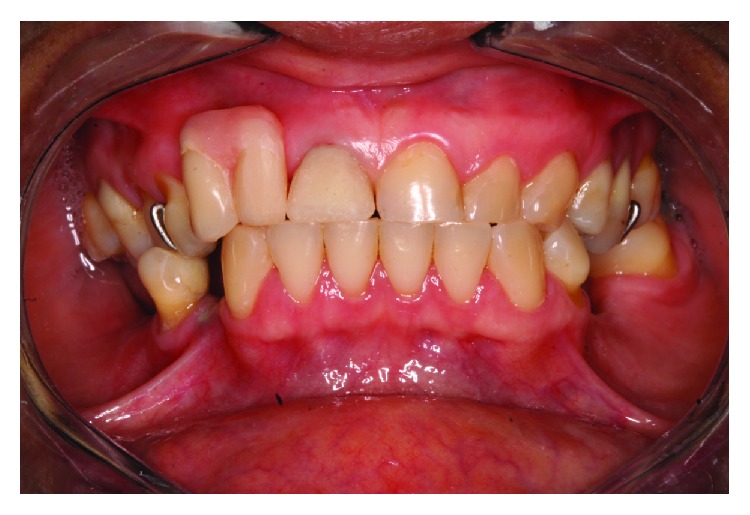
Temporary crown on teeth 11 adapted to the removable prosthesis.

A 44-year-old female patient reported to the Department of Oral and Maxillofacial Surgery, and Periodontology at the Dental School of Ribeirao Preto, University of São Paulo for a placement of implants. During the initial examination, it was observed that the right upper central incisor had an unsatisfactory temporary Richmond type crown that was changed by a metal core, post, and new temporary crown. The new temporary crown was fitted to the removable partial denture that was used by the patient (Figure 1). Surgical guides were made and the patient was referred for implant placement, in the region of teeth 12, 35, 36, 44, and 46.

**Figure 2:**
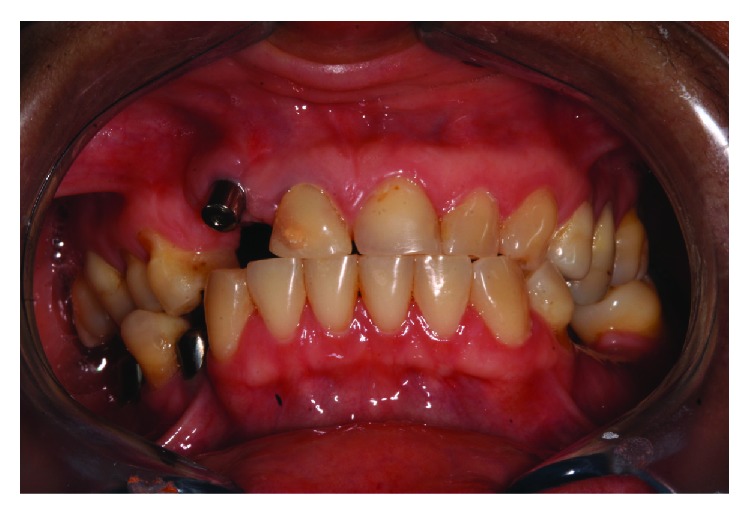
Poorly positioned implant.

After approximately 8 months, it was verified that the implant located in the anterior region was positioned further apical and buccal (Figure 2) making it impossible to fabricate a successful implant crown from an esthetic point of view.

**Figure 3:**
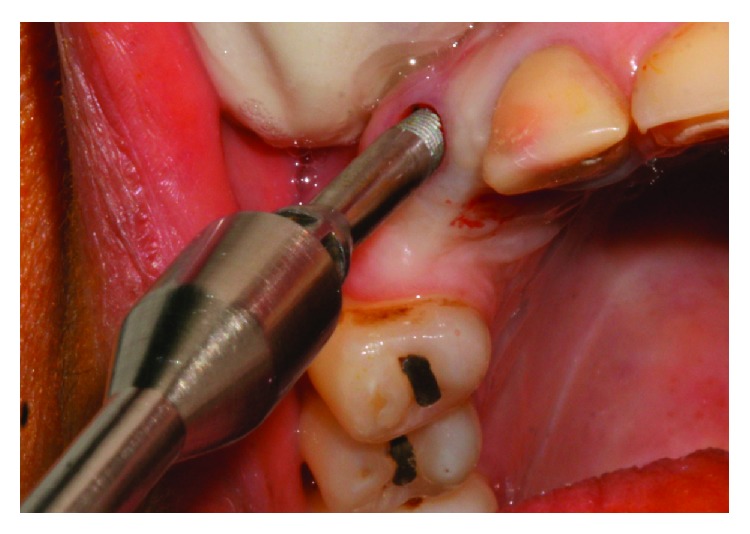
Removal of the implant with a retrieval tool.

**Figure 4:**
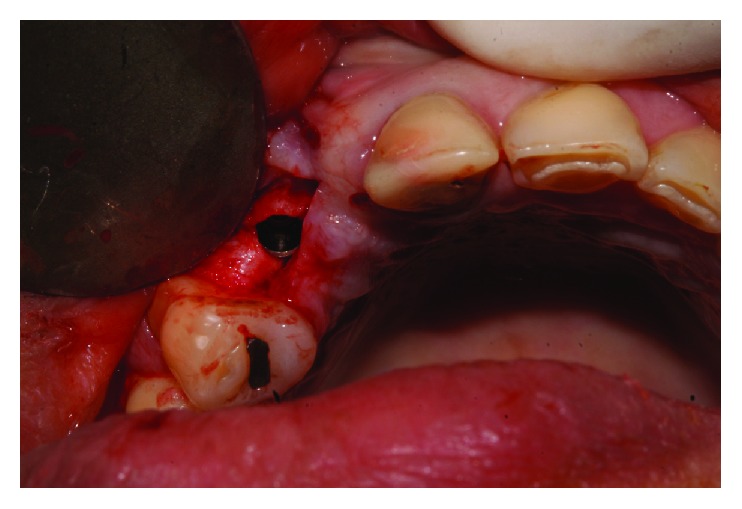
Installation of the new implant.

The implant was gradually removed using the implant removal instrument (Implant Retrieval Tool; Nobel Biocare), with antirotational movement (Figure 3). Following this surgical procedure, a new implant was installed (cone morse 3.5 × 11 mm, Conexão, Brazil), with a torque of 30 N, and positioned in the mesiodistal direction. Specifically, the implant was placed closer to tooth 14 in the buccal-palatine direction on the bone ridge (located around 1 mm for the palatal from an imaginary curvature passing through the buccal surfaces of the present teeth and 1 mm infraosseo in the coronoapical direction) (Figure 4).

**Figure 5:**
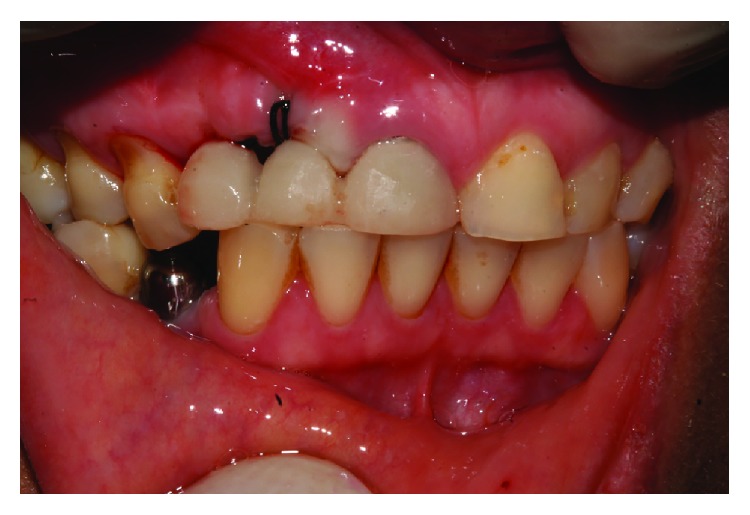
Installation of the temporary fixed partial denture.

After the surgery, the removable partial denture was removed. This was followed by the fabrication of a temporary fixed partial prosthesis with retention used during the osseointegration phase on teeth 11 and 14 (Figure 5): this was only for esthetic purpose. This was in addition to the installation of prostheses on the implants in the regions of teeth 35, 36, 44, and 46.

**Figure 6:**
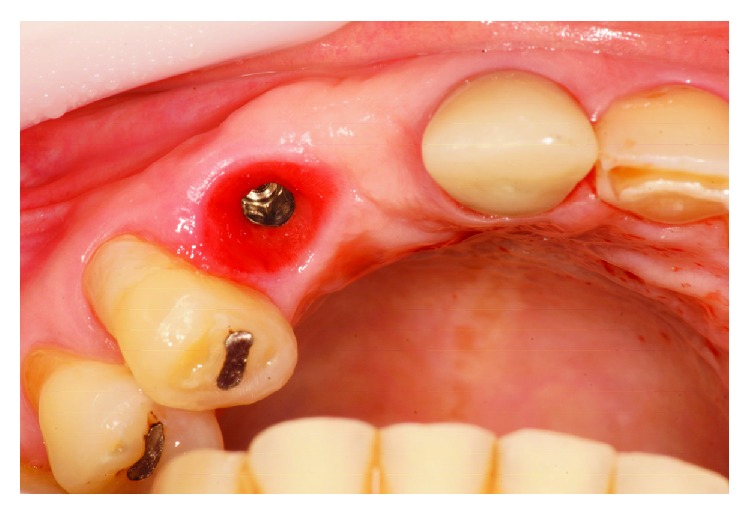
Occlusal view of the gingival conditioning.

**Figure 7:**
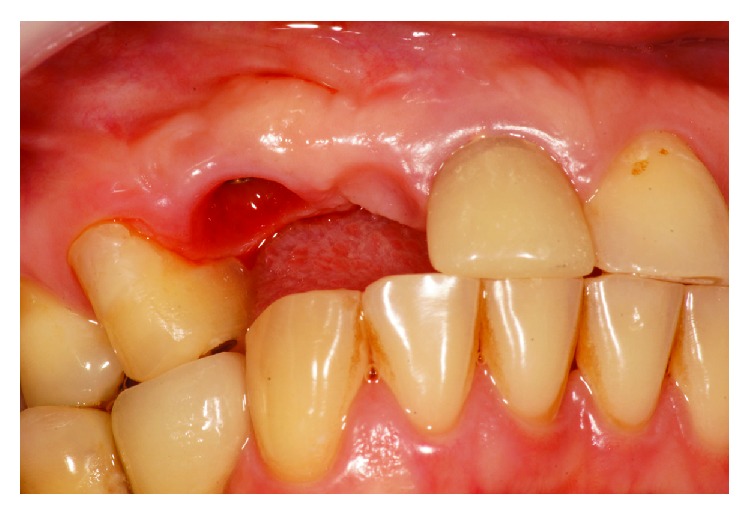
Buccal view of the gingival conditioning.

**Figure 8:**
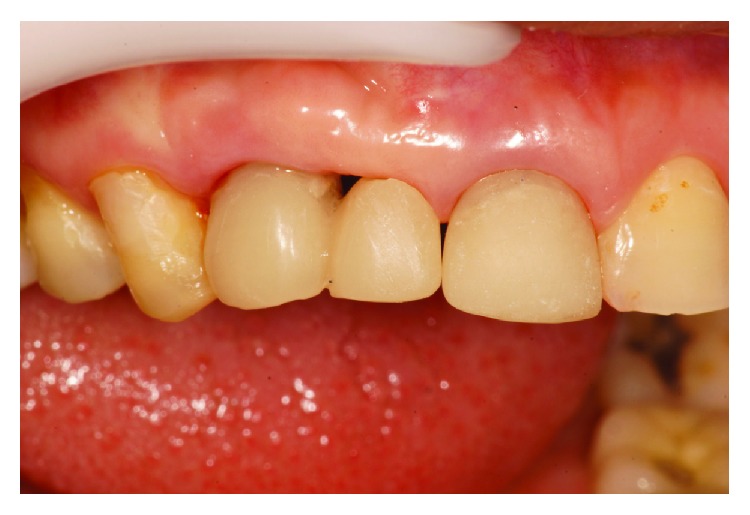
Presence of the black triangle before gingival conditioning.

**Figure 9:**
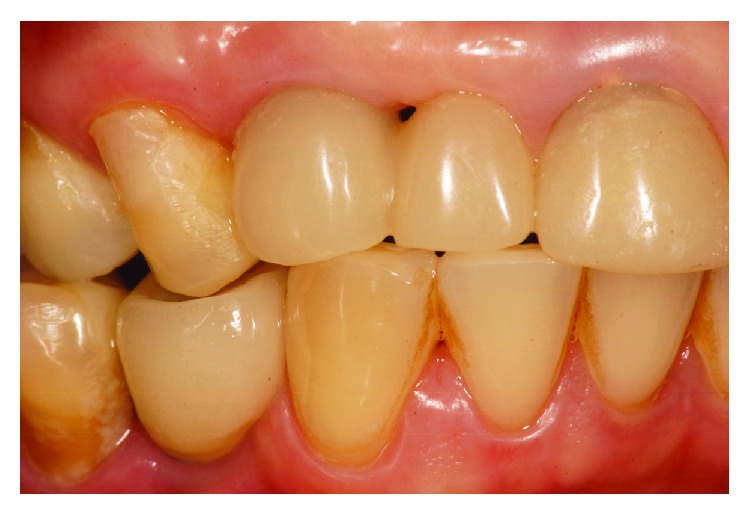
Gradual closing of the black triangle.

Immediately after the second stage surgery, the temporary fixed partial denture was performed using the healing cap as an abutment and a lateral incisor as a mesial cantilever. Following the rehabilitation procedures, this temporary fixed partial denture was changed to a provisional fixed prosthesis with the mesial cantilever screwed into the implant. During this phase, periodic acrylic resin implements were added to the temporary prosthesis to promote gingival conditioning (Figures 6 and 7) and black triangle closure (Figures 8 and 9) and also to improve gingival esthetics.

**Figure 10:**
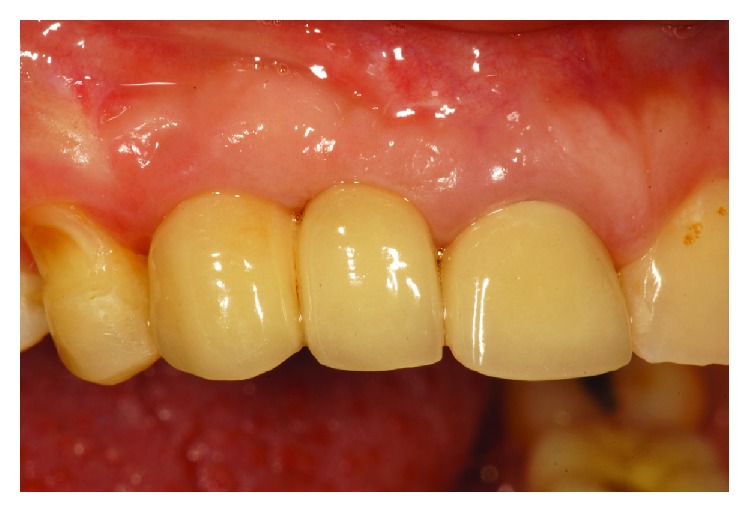
Final appearance of the implant prosthesis.

At the end of two months, the implant prosthesis with a mesial cantilever was installed (Figure 10). This case report was approved by the institution's Ethics Committee on Human Research and followed the ethical principles of the Declaration of Helsinki, in addition to complying with specific legislation.

## 3. Discussion

From an esthetic point of view, a misplaced implant, especially in the anterior region, generally results in an unsatisfactory prosthesis. Since esthetics is a primary requirement of the treatment regimen, this error is prevented through appropriate planning of the surgery for implant placement.

A typical successful course of treatment involves, but is not limited to, clinical exams, mounted casts in the semiadjustable articulator, a diagnostic waxing-up, radiographic images, surgical guides, and bone and/or soft tissue grafts which are part of a set of procedures that must be strictly followed [14].

The introduction of the cone beam computerized tomography (CBCT) in dentistry has made it possible to perform a precise preoperative evaluation of the implant sites and sophisticated surgical guide in dental implantology [8, 15]. The guided implant protocols have made the clinicians simplify their procedures starting from the diagnostic phase up to the realization of the final prosthetic restoration [7]. However, as these resources are not always available for a large part of the population, even with the best preparation and planning, undesirable occurrences can result into a detriment or can even render the treatment impracticable [10], a development which, without a doubt, will likely generate dissatisfaction, frustration, the necessity of other surgery, and loss of time for the patient.

Until recently, the removal of an implant resulted in a heavy loss of bone tissue and the necessity of bone grafting procedures [16]. However, technological advancements have led to the development of instruments that facilitate implant removal via conservative and easy procedures [13, 17].

Some points are worth making for this report. In this case, despite the careful presurgical planning, complications during the surgery resulted in a case with no prosthetic solution, even if using angled implant abutment due to the buccal positioning of the implant. The angled abutment presented a metallic platform wider than the straight one, thereby negatively affecting esthetics. This condition could have been avoided if the positioning of the implant had been 1 to 2 mm more palatal from an imaginary line passing through the buccal surfaces of the other teeth [1, 6]. This would facilitate prosthetic procedures, preserve facial esthetics, and conserve bone tissue on the buccal surfaces.

The apical placement of the implant is another deficient aspect of the case study, because it created a misalignment in relation to the natural teeth. Ideally, it should have been placed 3 to 4 mm apical to the cementoenamel junction of the adjacent teeth [6] to achieve the esthetic goal. This would have resulted in a smooth gingival contour, without abrupt changes in tissue height [1]. In addition, this implant positioning would necessarily require a very long clinical crown, disproportionate to adjacent teeth and visible during the patient's smile.

Therefore, due to the poor prognosis, the treatment option was to remove the implant and carefully replace it with a new one that was three-dimensionally well positioned, enabling the prosthetic rehabilitation. Among the various possibilities [9, 11, 12], the selected technique allowed the implant to be removed quickly, atraumatically, and without the need for incisions or manipulation of bone tissue. Considering the prosthetic planning, even if the implant had been adequately placed in the apicocoronal and buccopalatal direction, the prosthetic resolution for the implant in the site of tooth 12 would be a distal cantilever of tooth 13. This is a controversial option from a mechanical and biological point of view [18]. Another option could have been a fixed partial denture using the implant abutment in the site of tooth 12 and a tooth abutment on tooth 14, which was also not considered as the first choice [19, 20]. The surgeon was then asked to place the new implant in the mesiodistal direction, closest to tooth 14, in the position of tooth 13, to serve as an abutment for a mesial cantilever prosthesis in the site of tooth 12, avoiding the trauma caused by the placement of another implant [21]. In addition, studies have shown that the lateral incisor had the thinnest alveolar ridge compared to the central incisor and cuspid, probably due to the presence of the lateral fossa which creates the buccal concavity adjacent to the lateral incisor [15].

Regarding the immediate provisionalization, because the temporary prosthesis was retained only by tooth 11, its main objective was the patient's satisfaction with the esthetics during the period of osseointegration [1, 22] despite some benefit in preserving the tissue integrity. It is important to emphasize that this temporary restoration presented no functional characteristic. In other words, occlusal loads were not incident on it, and after second stage surgery, the prosthesis was adjusted and additionally retained on the healing cap.

After the osseointegration period and during the prosthetic treatment, the alignment of the gingival height and adequate conformation of the gingival papilla between the first right upper premolar and the implant were verified. According to Buser et al. [1], the harmony of the gingival margins around the implant and the adjacent tooth requires sufficient height and thickness of the bone, mainly in the buccal side, which was verified in the present case. However, in the cantilever region, there was a need for tissue conditioning by the gradual addition of self-curing resin, giving an oval characteristic to the pontic of the provisional prosthetic restoration to conform to the underlying soft tissue. As bone and connective tissue grafts were not performed, the shape of the interproximal papilla was not completely reestablished. It was necessary to modify the interproximal contact from point to facet of contact with the goal of reducing the black triangle and improving the esthetics of the region, as suggested by Jivraj and Chee [3].

The maxillary anterior region may be the implant site that requires the most rigorous preoperative assessment [15]. In the present case, the esthetic result was satisfactory, mainly when compared with the initial aspect, even with no bone or soft tissue graft procedures. Two other factors should be considered in a treatment and not only the result of the treatment itself [7]: (1) the economic condition of the patient because of the cost of sophisticated procedures and exams, and (2) the patient satisfaction with the final result of the treatment. Still, according to Stajčić et al. [4], studies have shown that there is no definitive evidence in improving esthetics with the use of bone and soft tissue grafts, justifying the option of the selected treatment. Furthermore, in the present case, the economic condition of the patient was considered as important.

Another important aspect was the position of the new implant, in the mesiodistal direction closest to tooth 14. This allowed the placement of a mesial cantilever that is more favorable to the occlusal loads than a distal cantilever [3], and the screw-type prosthesis is more easily retrievable than the cemented type and, therefore, technical and sometimes biological complications can be treated more easily [23].

## 4. Conclusion

Regardless of the cause, when an implant is not well positioned, prosthetic rehabilitation may not be mechanically, functionally, and esthetically adequate, and may even be impracticable to perform. In the case presented here, the removal of the implant and new surgical and prosthetic planning were necessary. Despite the esthetic limitations of the resulting prosthetic rehabilitation, considering that no bone and soft tissue grafting has been performed, it was in agreement with the expectations of the patient and the prosthodontist.

## References

[1] Buser D., Martin W., Belser U. C. (2004). Optimizing esthetics for implant restorations in the anterior maxilla: anatomic and surgical considerations. *International Journal of Oral & Maxillofacial Implants*.

[2] Moráguez O. D., Vailati F., Belser U. C. (2015). Malpositioned implants in the anterior maxilla: a novel restorative approach to reestablish peri-implant tissue health and acceptable esthetics. Part II: case report and discussion. *Journal of Esthetic Dentistry*.

[3] Jivraj S., Chee W. (2006). Treatment planning of implants in the aesthetic zone. *British Dental Journal*.

[4] Stajčić Z., Stojčev Stajčić L. J., Kalanović M., Đinić A., Divekar N., Rodić M. (2016). Removal of dental implants: review of five different techniques. *International Journal of Oral & Maxillofacial Surgery*.

[5] Kim Y., Kim B., Lee H., Hwang J., Yun P. (2010). Surgical repositioning of an unrestorable implant using a trephine bur: a case report. *International Journal of Periodontics & Restorative Dentistry*.

[6] Jung S.-R., Bashutski J. D., Linebaugh M. L. (2013). Application of modified bony lid technique to remove or replace compromised implants: case series. *Implant Dentistry*.

[7] Rumfola J. L., Andreana S., Colucci L., Tsay Y. (2013). Restoring unfavorably positioned implants in anterior maxilla: case report. *New York State Dental Journal*.

[8] Li C. H., Chou C. T. (2014). Bone sparing implant removal without trephine via internal separation of the titanium body with a carbide bur. *International Journal of Oral & Maxillofacial Surgery*.

[9] Chee W., Jivraj S. (2007). Failures in implant dentistry. *British Dental Journal*.

[10] Muroff F. I. (2003). Removal and replacement of a fractured dental implant: case report. *Implant Dentistry*.

[11] Lee J.-B. (2017). Selectable implant removal methods due to mechanical and biological failures. *Case Reports in Dentistry*.

[12] Romanos G. E., Gupta B., Eckert S. E. (2012). Distal cantilevers and implant dentistry. *International Journal of Oral & Maxillofacial Implants*.

[13] Schlumberger T. L., Bowley J. F., Maze G. I. (1998). Intrusion phenomenon in combination tooth-implant restorations: a review of the literature. *Journal of Prosthetic Dentistry*.

[14] Becker C. M., Kaiser D. A., Jones J. D. (2000). Guidelines for splinting implants. *Journal of Prosthetic Dentistry*.

[15] Levin L. (2008). Dealing with dental implant failures. *Journal of Applied Oral Science*.

[16] Conte G. J., Rhodes P., Richards D., Kao R. T. (2002). Considerations for anterior implant esthetics. *Journal of the California Dental Association*.

[17] Colombo M., Mangano C., Mijiritsky E., Krebs M., Hauschild U., Fortin T. (2017). Clinical applications and effectiveness of guided implant surgery: a critical review based on randomized controlled trials. *BMC Oral Health*.

[18] Testori T., Weinstein T., Scutella F., Wang H. L., Zucchelli G. (2018). Implant placement in the esthetic area: criteria for positioning single and multiple implants. *Periodontology 2000*.

[19] Mangano F. G., Mastrangelo P., Luongo F., Blay A., Tunchel S., Mangano C. (2017). Aesthetic outcome of immediately restored single implants placed in extraction sockets and healed sites of the anterior maxilla: a retrospective study on 103 patients with 3 years of follow-up. *Clinical Oral Implants Research*.

[20] Zhang W., Skrypczak A., Weltman R. (2015). Anterior maxilla alveolar ridge dimension and morphology measurement by cone beam computerized tomography (CBCT) for immediate implant treatment planning. *BMC Oral Health*.

[21] Khzam N., Arora H., Kim P., Fisher A., Mattheos N., Ivanovski S. (2015). Systematic review of soft tissue alterations and esthetic outcomes following immediate implant placement and restoration of single implants in the anterior maxilla. *Journal of Periodontology*.

[22] Van Nimwegen W. G., Raghoebar G. M., Tymstra N., Vissink A., Meijer H. J. A. (2017). How to treat two adjacent missing teeth with dental implants. A systematic review on single implant-supported two-unit cantilever FDP's and results of a 5-year prospective comparative study in the aesthetic zone. *Journal of Oral Rehabilitation*.

[23] Sailer I., Mühlemann S., Zwahlen M., Hämmerle C. H. F., Schneider D. (2012). Cemented and screw-retained implant reconstructions: a systematic review of the survival and complication rates. *Clinical Oral Implants Research*.

